# 1-(6-Chloro-1-methyl-1*H*-imidazo[4,5-*c*]pyridin-4-yl)-3-(2-chloro­phen­yl)urea

**DOI:** 10.1107/S1600536814000695

**Published:** 2014-01-18

**Authors:** Venkatesh B. Devaru, M. Vinduvahini, M. Madaiah, H. D. Revanasiddappa, H. C. Devarajegowda

**Affiliations:** a P. G. Department of Physics, LVD College, Raichur 584 103, Karnataka, India; b Department of Physics, Sri D Devaraja Urs Govt. First Grade College, Hunsur 571 105, Mysore District, Karnataka, India; c Department of Studies in Chemistry, Manasagangotri, University of Mysore, Mysore 570 006, Karnataka, India; d Department of Physics, Yuvaraja’s College (Constituent College), University of Mysore, Mysore 570 005, Karnataka, India

## Abstract

In the title compound, C_14_H_11_Cl_2_N_5_O, the plane of the 1*H*-imidazo[4,5-*c*]pyridine ring system [r.m.s. deviation = 0.087 (19) Å] makes a dihedral angle of 4.87 (10)° with the terminal phenyl ring. An intra­molecular N—H⋯N hydrogen bond stabilizes the mol­ecular conformation. In the crystal, N—H⋯O hydrogen bonds link the mol­ecules into inversion dimers. These dimers are connected by π–π inter­actions between imidazole rings [shortest centroid–centroid distance = 3.4443 (14) Å].

## Related literature   

For biological applications of imidazo­pyridines, see: Cappelli *et al.* (2006 ▶); Weier *et al.* (1994 ▶); Barraclough *et al.* (1990 ▶); Bavetsias *et al.* (2007 ▶); Cooper *et al.* (1992 ▶); Temple *et al.* (1987 ▶); Janssens *et al.* (1985 ▶); Kulkarni & Newman (2007 ▶). For a related structure, see: Kandri Rodi *et al.* (2013 ▶).
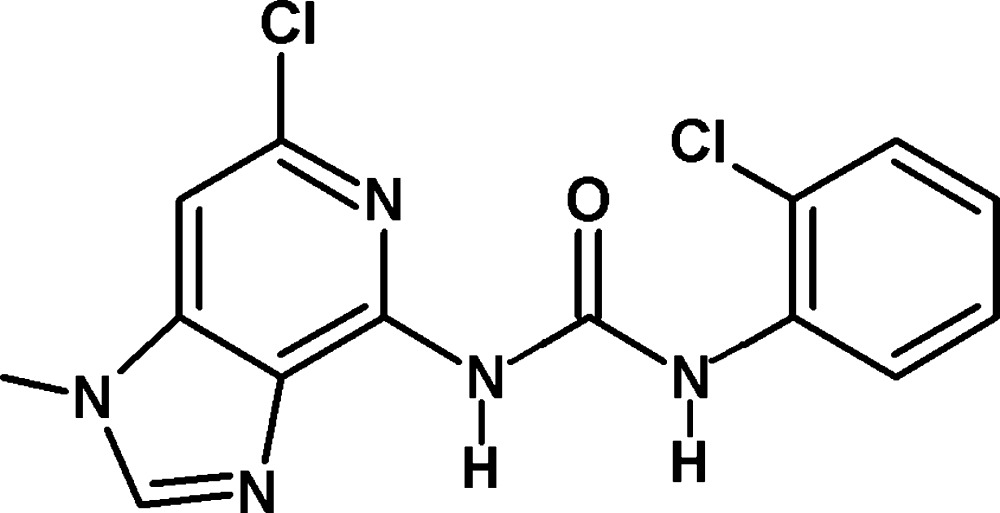


## Experimental   

### 

#### Crystal data  

C_14_H_11_Cl_2_N_5_O
*M* *_r_* = 336.18
Monoclinic, 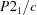

*a* = 8.9368 (3) Å
*b* = 17.2369 (4) Å
*c* = 10.3805 (3) Å
β = 114.216 (4)°
*V* = 1458.33 (7) Å^3^
*Z* = 4
Mo *K*α radiation
μ = 0.45 mm^−1^
*T* = 293 K
0.24 × 0.20 × 0.12 mm

#### Data collection  

Bruker SMART CCD area-detector diffractometer
Absorption correction: multi-scan (*SADABS*; Sheldrick, 2007 ▶) *T*_min_ = 0.770, *T*_max_ = 1.000
11425 measured reflections
2576 independent reflections
2175 reflections with *I* > 2σ(*I*)
*R*_int_ = 0.023

#### Refinement  

*R*[*F* ^2^ > 2σ(*F* ^2^)] = 0.039
*wR*(*F* ^2^) = 0.112
*S* = 1.06
2576 reflections
199 parameters
H-atom parameters constrained
Δρ_max_ = 0.38 e Å^−3^
Δρ_min_ = −0.28 e Å^−3^

### 

Data collection: *SMART* (Bruker, 2001 ▶); cell refinement: *SAINT* (Bruker, 2001 ▶); data reduction: *SAINT*; program(s) used to solve structure: *SHELXS97* (Sheldrick, 2008 ▶); program(s) used to refine structure: *SHELXL97* (Sheldrick, 2008 ▶); molecular graphics: *ORTEP-3 for Windows* (Farrugia, 2012 ▶); software used to prepare material for publication: *SHELXL97*.

## Supplementary Material

Crystal structure: contains datablock(s) I, global. DOI: 10.1107/S1600536814000695/bt6956sup1.cif

Structure factors: contains datablock(s) I. DOI: 10.1107/S1600536814000695/bt6956Isup2.hkl

Supporting information file. DOI: 10.1107/S1600536814000695/bt6956Isup3.cml

CCDC reference:

Additional supporting information:  crystallographic information; 3D view; checkCIF report

## Figures and Tables

**Table 1:** Hydrogen-bond geometry (Å, °)

*D*—H⋯*A*	*D*—H	H⋯*A*	*D*⋯*A*	*D*—H⋯*A*
N7—H7⋯O3^i^	0.86	2.07	2.862 (3)	153
N8—H8⋯N6	0.86	2.03	2.723 (2)	136

Symmetry code: (i) 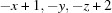.

**Fig. 1.**
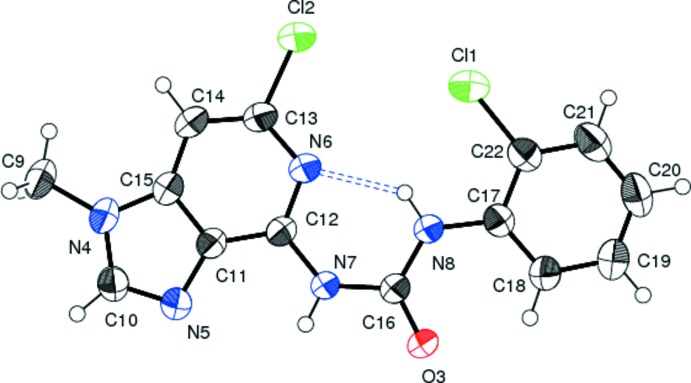
The molecular structure of the title compound. Displacement ellipsoids are drawn at the 50% probability level. Hydrogen atoms are shown as spheres of arbitrary radius. Hydrogen bonds are shown as open dashed bonds.

**Fig. 2.**
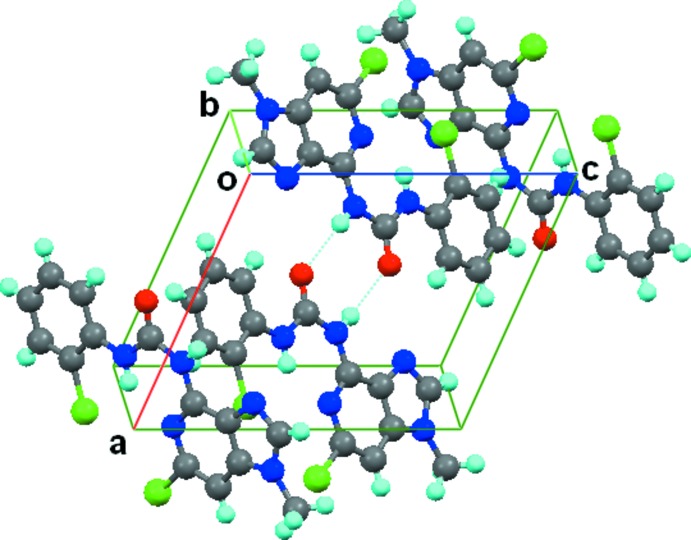
The packing of the title compound. Intermolecular hydrogen bonds are shown as dashed lines.

## supplementary crystallographic information

### 1. Comment

The identification of chemotherapeutic targets could lead to new therapeutic approaches and may be a key for the discovery of really effective drugs. The imidazopyridine (Cappelli *et al.*, 2006; Weier *et al.*, 1994; Barraclough *et al.*, 1990; Bavetsias *et al.*, 2007) moieties are important pharmacophores, which have proven to be useful for a number of biologically relevant targets. The compounds derived from the imidazopyridine system have recently been evaluated as antagonists of various biological receptors, including angiotensin-II and platelet activating factor (Cooper *et al.*, 1992). Substituted imidazo[4,5]pyridines have also been tested for their potential as anticancer (Temple *et al.*, 1987) and selective antihistamine (H1) agents (Janssens *et al.*, 1985). Imidazo[4,5]pyridine derivatives were also reported as inhibitors of Mitogen and stress-activated protein kinases and Aurora kinases (Kulkarni & Newman, 2007). The bond lengths and bond angles are good agreement with a related structure (Kandri Rodi *et al.*, 2013)

The asymmetric unit of 1-(6-chloro-1-methyl-1*H*-imidazo [4,5-*c*]pyridin-4-yl)-3-(2-chlorophenyl)urea is shown in Fig. 1. The 1*H*-imidazo[4,5-*c*]pyridine ring (N4/N5/N6/C10–C15) system makes a dihedral angle of 4.87 (10)° with the terminal phenyl ring.

An intramolecular N-H···N hydrogen bond stabilizes the molecular conformation. Intermolecular N-H···O hydrogen bonds link the molecules to centrosymmetric dimers. These dimers are further connected by intermolecular π–π interactions between imidazole rings [shortest centroid–centroid distance = 3.4443 (14) Å]. A view of the crystal packing is given in Figure 2.

### 2. Experimental

A mixture of 2,4,6-trichloropyridene, methylamine in ethanol was heated and filtered to get pure product. To this sulfuric acid and fuming nitric acid was added, then it was stirred and cooled. A solution of iron powder and ammonium chloride in methanol/water was added and heated. Then triethylorthoformate in ethanol was added and continued the heating. Amination of reaction product was achieved by adding benzophenone imine, potassium carbonate, palladium complex, in dioxane, and then it was heated. The obtained product was dissolved in HCl, stirred, and concentrated *in vacuo* to give the product. A mixture of obtained product, sodium hydride, 6-chloro-1-methyl-1*H*-imidazol [4,5-*c*]pyridin-4-amine and carbonyl/sulfonyl chlorides in tetahydrofuran was stirred and concentrated *in vacuo* to give the expected products. After completion of each step of the reaction TLC was monitored. The compound is recrystallized by ethanol- chloroform mixture. Colourless needles of the title compound were grown from a mixed solution of Ethanol/Chloroform (V/V = 2/1) by slow evaporation at room temperature. Yield: 122 mg, 66.27%; m p: 380; IR cm-1 (KBr) 3431, 1669; Anal. Calcd for C_14_H_11_Cl_2_N_5_O C,50.02; H, 3.30; N, 20.83%; Found, C, 49.45; H, 3.25; N, 20.44%.

### 3. Refinement

All H atoms were positioned geometrically; N—H = 0.86 Å, C—H = 0.93 Å for aromatic H, and C—H = 0.96 Å for methyl H, and refined using a riding model with *U*_iso_(H) = 1.5*U*_eq_(C) for methyl H and *U*_iso_(H) = 1.2*U*_eq_(C) for all other H.

### Crystal data

**Table:**

C_14_H_11_Cl_2_N_5_O	*F*(000) = 688
*M**_r_* = 336.18	*D*_x_ = 1.531 Mg m^−^^3^
Monoclinic, *P*2_1_/*c*	Melting point: 380 K
Hall symbol: -P 2ybc	Mo *K*α radiation, λ = 0.71073 Å
*a* = 8.9368 (3) Å	Cell parameters from 2576 reflections
*b* = 17.2369 (4) Å	θ = 2.4–25.0°
*c* = 10.3805 (3) Å	µ = 0.45 mm^−^^1^
β = 114.216 (4)°	*T* = 293 K
*V* = 1458.33 (7) Å^3^	Plate, colourless
*Z* = 4	0.24 × 0.20 × 0.12 mm

### Data collection

**Table:**

Bruker SMART CCD area-detector diffractometer	2576 independent reflections
Radiation source: fine-focus sealed tube	2175 reflections with *I* > 2σ(*I*)
Graphite monochromator	*R*_int_ = 0.023
ω and φ scans	θ_max_ = 25.0°, θ_min_ = 2.4°
Absorption correction: multi-scan (*SADABS*; Sheldrick, 2007)	*h* = −10→10
*T*_min_ = 0.770, *T*_max_ = 1.000	*k* = −20→20
11425 measured reflections	*l* = −12→12

### Refinement

**Table:**

Refinement on *F*^2^	Primary atom site location: structure-invariant direct methods
Least-squares matrix: full	Secondary atom site location: difference Fourier map
*R*[*F*^2^ > 2σ(*F*^2^)] = 0.039	Hydrogen site location: inferred from neighbouring sites
*wR*(*F*^2^) = 0.112	H-atom parameters constrained
*S* = 1.06	*w* = 1/[σ^2^(*F*_o_^2^) + (0.0553*P*)^2^ + 0.5893*P*] where *P* = (*F*_o_^2^ + 2*F*_c_^2^)/3
2576 reflections	(Δ/σ)_max_ = 0.001
199 parameters	Δρ_max_ = 0.38 e Å^−^^3^
0 restraints	Δρ_min_ = −0.28 e Å^−^^3^

### Special details

**Table:**

Experimental. IR cm-1 (KBr) 3431, 1669; Anal. Calcd for C_14_H_11_Cl_2_N_5_O C,50.02; H, 3.30; N, 20.83%; Found, C, 49.45; H, 3.25; N, 20.44%.
Geometry. All s.u.'s (except the s.u. in the dihedral angle between two l.s. planes) are estimated using the full covariance matrix. The cell s.u.'s are taken into account individually in the estimation of s.u.'s in distances, angles and torsion angles; correlations between s.u.'s in cell parameters are only used when they are defined by crystal symmetry. An approximate (isotropic) treatment of cell s.u.'s is used for estimating s.u.'s involving l.s. planes.
Refinement. Refinement of *F*^2^ against ALL reflections. The weighted *R*-factor *wR* and goodness of fit *S* are based on *F*^2^, conventional *R*-factors *R* are based on *F*, with *F* set to zero for negative *F*^2^. The threshold expression of *F*^2^ > 2σ(*F*^2^) is used only for calculating *R*-factors(gt) *etc*. and is not relevant to the choice of reflections for refinement. *R*-factors based on *F*^2^ are statistically about twice as large as those based on *F*, and *R*- factors based on ALL data will be even larger.

### Fractional atomic coordinates and isotropic or equivalent isotropic displacement parameters (Å^2^)

**Table:**

	*x*	*y*	*z*	*U*_iso_*/*U*_eq_	
Cl1	0.98140 (8)	0.21079 (4)	0.85698 (7)	0.0611 (2)	
Cl2	1.28185 (8)	0.14285 (4)	1.18265 (7)	0.0693 (2)	
O3	0.50876 (19)	0.04206 (11)	0.86633 (16)	0.0599 (5)	
N4	1.1433 (2)	−0.06979 (11)	1.47101 (19)	0.0457 (5)	
N5	0.8783 (2)	−0.07679 (11)	1.31848 (19)	0.0474 (5)	
N6	1.0050 (2)	0.07252 (10)	1.12745 (18)	0.0424 (4)	
N7	0.7418 (2)	0.01864 (11)	1.05733 (18)	0.0447 (5)	
H7	0.6847	−0.0136	1.0818	0.054*	
N8	0.7379 (2)	0.10758 (10)	0.88673 (17)	0.0389 (4)	
H8	0.8420	0.1107	0.9357	0.047*	
C9	1.2941 (3)	−0.08685 (16)	1.5946 (2)	0.0587 (6)	
H9A	1.3816	−0.0553	1.5925	0.088*	
H9B	1.3215	−0.1406	1.5937	0.088*	
H9C	1.2783	−0.0758	1.6789	0.088*	
C10	0.9933 (3)	−0.10224 (14)	1.4362 (2)	0.0502 (6)	
H10	0.9738	−0.1398	1.4919	0.060*	
C11	0.9608 (3)	−0.02290 (12)	1.2725 (2)	0.0386 (5)	
C12	0.9041 (2)	0.02356 (12)	1.1512 (2)	0.0379 (5)	
C13	1.1616 (3)	0.07603 (13)	1.2225 (2)	0.0451 (5)	
C14	1.2320 (3)	0.03333 (13)	1.3434 (2)	0.0461 (5)	
H14	1.3418	0.0380	1.4053	0.055*	
C15	1.1242 (3)	−0.01774 (12)	1.3656 (2)	0.0397 (5)	
C16	0.6546 (3)	0.05620 (13)	0.9311 (2)	0.0408 (5)	
C17	0.6706 (3)	0.15605 (11)	0.7687 (2)	0.0389 (5)	
C18	0.5051 (3)	0.15738 (14)	0.6761 (2)	0.0472 (5)	
H18	0.4318	0.1235	0.6897	0.057*	
C19	0.4496 (3)	0.20893 (14)	0.5640 (3)	0.0545 (6)	
H19	0.3389	0.2093	0.5032	0.065*	
C20	0.5541 (4)	0.25923 (15)	0.5406 (3)	0.0621 (7)	
H20	0.5148	0.2933	0.4646	0.075*	
C21	0.7172 (4)	0.25902 (14)	0.6304 (3)	0.0599 (7)	
H21	0.7893	0.2929	0.6152	0.072*	
C22	0.7742 (3)	0.20852 (12)	0.7430 (2)	0.0444 (5)	

### Atomic displacement parameters (Å^2^)

**Table:**

	*U*^11^	*U*^22^	*U*^33^	*U*^12^	*U*^13^	*U*^23^
Cl1	0.0526 (4)	0.0597 (4)	0.0696 (4)	−0.0158 (3)	0.0237 (3)	−0.0005 (3)
Cl2	0.0440 (4)	0.0822 (5)	0.0688 (4)	−0.0224 (3)	0.0101 (3)	0.0153 (3)
O3	0.0347 (9)	0.0818 (12)	0.0484 (9)	−0.0163 (8)	0.0022 (7)	0.0204 (8)
N4	0.0397 (10)	0.0505 (11)	0.0404 (10)	0.0087 (8)	0.0100 (8)	0.0054 (8)
N5	0.0400 (10)	0.0526 (11)	0.0467 (11)	0.0006 (9)	0.0147 (9)	0.0106 (9)
N6	0.0363 (10)	0.0455 (10)	0.0408 (10)	−0.0063 (8)	0.0112 (8)	−0.0004 (8)
N7	0.0341 (10)	0.0529 (11)	0.0387 (10)	−0.0093 (8)	0.0065 (8)	0.0110 (8)
N8	0.0336 (9)	0.0449 (10)	0.0351 (9)	−0.0055 (8)	0.0111 (7)	0.0019 (8)
C9	0.0453 (14)	0.0688 (16)	0.0477 (14)	0.0113 (12)	0.0047 (11)	0.0098 (12)
C10	0.0472 (13)	0.0525 (13)	0.0499 (13)	0.0054 (11)	0.0188 (11)	0.0130 (11)
C11	0.0343 (11)	0.0416 (11)	0.0379 (11)	0.0012 (9)	0.0126 (9)	0.0003 (9)
C12	0.0327 (11)	0.0422 (11)	0.0362 (11)	−0.0005 (9)	0.0114 (9)	−0.0016 (9)
C13	0.0357 (11)	0.0494 (13)	0.0465 (12)	−0.0066 (10)	0.0131 (10)	−0.0024 (10)
C14	0.0315 (11)	0.0536 (13)	0.0450 (12)	−0.0006 (10)	0.0074 (10)	−0.0022 (10)
C15	0.0378 (11)	0.0415 (11)	0.0365 (11)	0.0041 (9)	0.0119 (9)	−0.0026 (9)
C16	0.0363 (12)	0.0457 (12)	0.0363 (11)	−0.0043 (9)	0.0108 (9)	0.0019 (9)
C17	0.0470 (12)	0.0365 (11)	0.0353 (10)	−0.0009 (9)	0.0191 (10)	−0.0032 (9)
C18	0.0472 (13)	0.0524 (13)	0.0402 (12)	−0.0004 (11)	0.0162 (10)	0.0034 (10)
C19	0.0591 (15)	0.0563 (15)	0.0422 (13)	0.0083 (12)	0.0146 (12)	0.0050 (11)
C20	0.084 (2)	0.0523 (14)	0.0493 (14)	0.0061 (14)	0.0261 (14)	0.0143 (12)
C21	0.0801 (19)	0.0470 (14)	0.0592 (16)	−0.0078 (13)	0.0351 (15)	0.0056 (12)
C22	0.0544 (14)	0.0394 (11)	0.0433 (12)	−0.0050 (10)	0.0239 (11)	−0.0041 (9)

### Geometric parameters (Å, º)

**Table:**

Cl1—C22	1.741 (2)	C9—H9C	0.9600
Cl2—C13	1.737 (2)	C10—H10	0.9300
O3—C16	1.221 (2)	C11—C15	1.384 (3)
N4—C10	1.357 (3)	C11—C12	1.399 (3)
N4—C15	1.371 (3)	C13—C14	1.366 (3)
N4—C9	1.460 (3)	C14—C15	1.391 (3)
N5—C10	1.309 (3)	C14—H14	0.9300
N5—C11	1.387 (3)	C17—C18	1.395 (3)
N6—C12	1.330 (3)	C17—C22	1.396 (3)
N6—C13	1.343 (3)	C18—C19	1.384 (3)
N7—C12	1.379 (3)	C18—H18	0.9300
N7—C16	1.381 (3)	C19—C20	1.366 (4)
N7—H7	0.8600	C19—H19	0.9300
N8—C16	1.353 (3)	C20—C21	1.371 (4)
N8—C17	1.399 (3)	C20—H20	0.9300
N8—H8	0.8600	C21—C22	1.377 (3)
C9—H9A	0.9600	C21—H21	0.9300
C9—H9B	0.9600		
			
C10—N4—C15	105.76 (18)	C14—C13—Cl2	118.66 (17)
C10—N4—C9	127.3 (2)	C13—C14—C15	113.8 (2)
C15—N4—C9	127.0 (2)	C13—C14—H14	123.1
C10—N5—C11	102.76 (18)	C15—C14—H14	123.1
C12—N6—C13	118.28 (18)	N4—C15—C11	105.38 (18)
C12—N7—C16	131.50 (18)	N4—C15—C14	132.7 (2)
C12—N7—H7	114.3	C11—C15—C14	121.92 (19)
C16—N7—H7	114.3	O3—C16—N8	123.74 (19)
C16—N8—C17	126.16 (18)	O3—C16—N7	119.21 (19)
C16—N8—H8	116.9	N8—C16—N7	117.06 (18)
C17—N8—H8	116.9	C18—C17—C22	117.2 (2)
N4—C9—H9A	109.5	C18—C17—N8	124.54 (19)
N4—C9—H9B	109.5	C22—C17—N8	118.3 (2)
H9A—C9—H9B	109.5	C19—C18—C17	120.2 (2)
N4—C9—H9C	109.5	C19—C18—H18	119.9
H9A—C9—H9C	109.5	C17—C18—H18	119.9
H9B—C9—H9C	109.5	C20—C19—C18	121.4 (3)
N5—C10—N4	115.0 (2)	C20—C19—H19	119.3
N5—C10—H10	122.5	C18—C19—H19	119.3
N4—C10—H10	122.5	C19—C20—C21	119.4 (2)
C15—C11—N5	111.13 (18)	C19—C20—H20	120.3
C15—C11—C12	118.69 (19)	C21—C20—H20	120.3
N5—C11—C12	130.18 (19)	C20—C21—C22	119.9 (2)
N6—C12—N7	120.29 (18)	C20—C21—H21	120.0
N6—C12—C11	120.47 (19)	C22—C21—H21	120.0
N7—C12—C11	119.24 (18)	C21—C22—C17	121.9 (2)
N6—C13—C14	126.8 (2)	C21—C22—Cl1	118.86 (18)
N6—C13—Cl2	114.53 (16)	C17—C22—Cl1	119.23 (17)
			
C11—N5—C10—N4	0.0 (3)	C12—C11—C15—N4	−179.29 (18)
C15—N4—C10—N5	0.2 (3)	N5—C11—C15—C14	−179.01 (19)
C9—N4—C10—N5	−179.6 (2)	C12—C11—C15—C14	1.4 (3)
C10—N5—C11—C15	−0.2 (2)	C13—C14—C15—N4	−179.8 (2)
C10—N5—C11—C12	179.3 (2)	C13—C14—C15—C11	−0.7 (3)
C13—N6—C12—N7	179.27 (19)	C17—N8—C16—O3	5.1 (3)
C13—N6—C12—C11	0.0 (3)	C17—N8—C16—N7	−174.47 (18)
C16—N7—C12—N6	1.5 (4)	C12—N7—C16—O3	−179.5 (2)
C16—N7—C12—C11	−179.2 (2)	C12—N7—C16—N8	0.1 (4)
C15—C11—C12—N6	−1.1 (3)	C16—N8—C17—C18	−1.4 (3)
N5—C11—C12—N6	179.5 (2)	C16—N8—C17—C22	176.71 (19)
C15—C11—C12—N7	179.66 (19)	C22—C17—C18—C19	0.3 (3)
N5—C11—C12—N7	0.2 (3)	N8—C17—C18—C19	178.4 (2)
C12—N6—C13—C14	0.8 (4)	C17—C18—C19—C20	0.2 (4)
C12—N6—C13—Cl2	−179.04 (15)	C18—C19—C20—C21	−0.3 (4)
N6—C13—C14—C15	−0.5 (3)	C19—C20—C21—C22	−0.2 (4)
Cl2—C13—C14—C15	179.40 (16)	C20—C21—C22—C17	0.8 (4)
C10—N4—C15—C11	−0.3 (2)	C20—C21—C22—Cl1	−178.9 (2)
C9—N4—C15—C11	179.5 (2)	C18—C17—C22—C21	−0.8 (3)
C10—N4—C15—C14	178.9 (2)	N8—C17—C22—C21	−179.0 (2)
C9—N4—C15—C14	−1.3 (4)	C18—C17—C22—Cl1	178.84 (16)
N5—C11—C15—N4	0.3 (2)	N8—C17—C22—Cl1	0.6 (3)

### Hydrogen-bond geometry (Å, º)

**Table:**

*D*—H···*A*	*D*—H	H···*A*	*D*···*A*	*D*—H···*A*
N7—H7···O3^i^	0.86	2.07	2.862 (3)	153
N8—H8···N6	0.86	2.03	2.723 (2)	136

Symmetry code: (i) −*x*+1, −*y*, −*z*+2.
